# Spectral domain optical coherence tomography findings in acute syphilitic posterior placoid chorioretinitis

**DOI:** 10.1186/1869-5760-4-2

**Published:** 2014-01-27

**Authors:** Bryn M Burkholder, Theresa G Leung, Trucian A Ostheimer, Nicholas J Butler, Jennifer E Thorne, James P Dunn

**Affiliations:** 1 The Wilmer Eye Institute, The Johns Hopkins University School of Medicine, 1800 Orleans St. Woods 476, Baltimore, MD 21287, USA; 2 The Bloomberg School of Public Health, The Johns Hopkins University, 1800 Orleans St. Woods 476, Baltimore, MD 21287, USA

**Keywords:** Syphilis, Chorioretinis, Optical coherence tomography, Retinal pigment epithelium, Photoreceptor

## Abstract

**Background:**

We describe the spectral domain optical coherence tomography (SD-OCT) findings in three patients with acute syphilitic posterior placoid chorioretinitis (ASPPC). The SD-OCT images demonstrate the pathologic changes in ASPPC with a high level of anatomic detail and may provide information about the pathophysiology of the disease.

**Findings:**

We report a series of three consecutive patients seen at the Wilmer Eye Institute in 2012 and 2013 who presented with clinical and laboratory findings consistent with a diagnosis of unilateral ASPPC. Two of the three patients had HIV co-infection with good immune recovery. SD-OCT images from their initial (pre-treatment) presentation demonstrated thickening and hyperreflective nodularity of the choroid-retinal pigment epithelium (RPE) complex, with focal disruption of the overlying photoreceptor inner segment-outer segment junction in the areas corresponding to the retinal lesions seen on clinical examination. These changes improved with intravenous antibiotic treatment over a 3-month period of follow-up.

**Conclusions:**

SD-OCT imaging in ASPPC demonstrates reversible, focal thickening, and nodularity of the RPE with disruption of the overlying photoreceptor inner segment-outer segment junction. We believe that these SD-OCT images support the concept that ASPPC involves an inflammatory process at the level of the choroid-RPE with resultant structural and functional changes in the retinal photoreceptors. Further study with OCT imaging may be helpful in better understanding this disease.

## Findings

### Introduction

Acute syphilitic posterior placoid chorioretinitis (ASPPC) is a distinct clinical presentation of ocular syphilis, characterized by large, yellow-white geographic lesions involving the macula at the level of the outer retina/retinal pigment epithelium (RPE) [1,2]. The clinical, angiographic, and autofluorescence findings have been described [1-7]. High-resolution spectral domain optical coherence tomography (SD-OCT) imaging allows the evaluation of ASPPC with a new level of anatomic detail and may provide more information about the pathophysiology of this disease. We describe here the SD-OCT findings in three patients with ASPPC.

## Methods

Three patients with ASPPC, based on clinical characteristics and positive serologic treponemal testing, were identified from the records from the Division of Ocular Immunology in the Wilmer Eye Institute. SD-OCT (Spectralis; Heidelberg Engineering, Heidelberg, Germany) macular images from these patients were reviewed. Institutional Review Board (IRB)/Ethics Committee approval was obtained.

## Results

**Figure 1:**
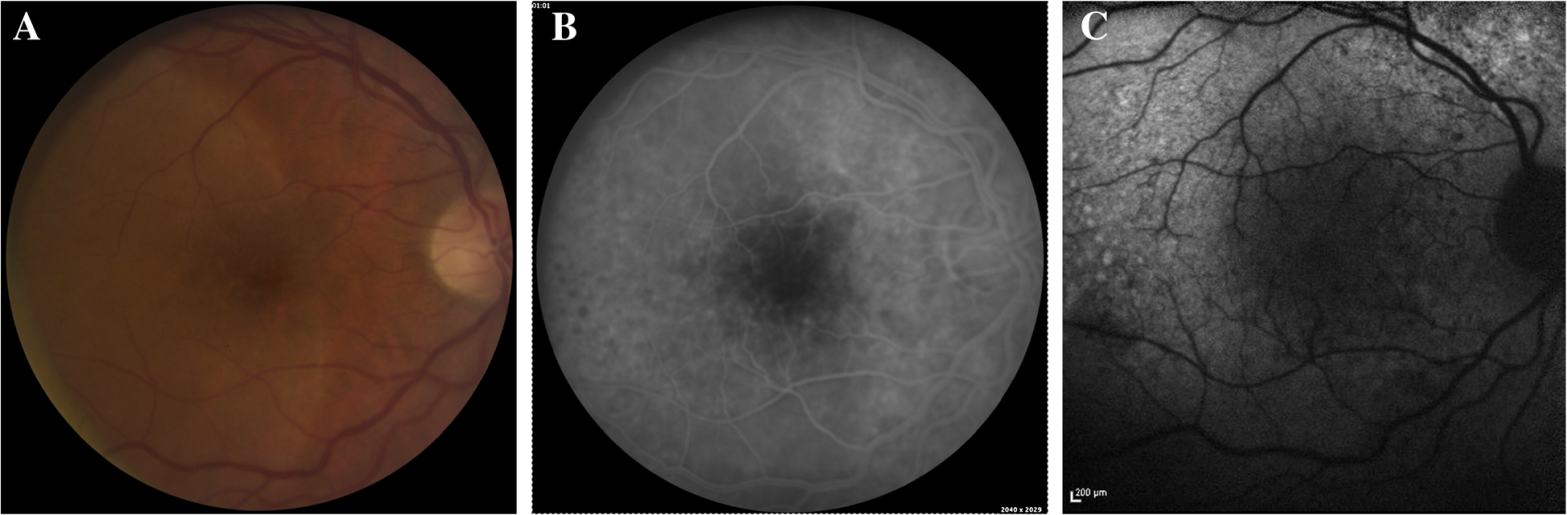
**Patient 1, pretreatment fundus imaging of the affected eye. (A)** Fundus photograph demonstrating placoid white retinal lesion. **(B)** Early phase fluorescein angiogram with mottled hypo- and hyperfluorescent spots (leopard spotting). **(C)** Fundus autofluoresence image with punctate areas of hyperautofluorescence in the temporal macula.

Patient 1 was a 47-year-old Caucasian male who presented with a central scotoma in the right eye. Corrected acuity measured 20/50 in the right eye (RE) and 20/20 in the left eye (LE) (Table 1). No inflammatory cells were noted in the anterior chamber or vitreous. Fundus examination showed a broad placoid area of retinal whitening in the macula of the RE (Figure 1). The treponemal test was positive, and the rapid plasma reagin (RPR) titer was 1:128. HIV antibody testing was negative. He received 14 days of intravenous penicillin G (24 million units/day), and vision improved to 20/20 in the right eye 3 months after treatment.

**Table 1:** Patient characteristics

**Patient**	**Gender**	**Age (years)**	**Ethnicity**	**HIV antibody**	**CD4 count (cells/μL)**	**FTA-ABS**^**a**^	**RPR**^**b **^**titer**	**Affected eye**	**Treatment**	**Pre-treatment visual acuity**	**Post-treatment visual acuity**
1	Male	47	Caucasian	Negative	n/a	Positive	1:128	Right	Intravenous penicillin	20/50	20/20
2	Male	60	Caucasian	Positive	600	Positive	Nonreactive	Right	Intravenous ceftriaxone	20/63	20/25
3	Male	50	Caucasian	Positive	830	Positive	1:4,096	Left	Intravenous penicillin	20/60	20/25

^a^Fluorescent treponemal antibody-absorption; ^b^rapid plasma reagin.

**Figure 2:**
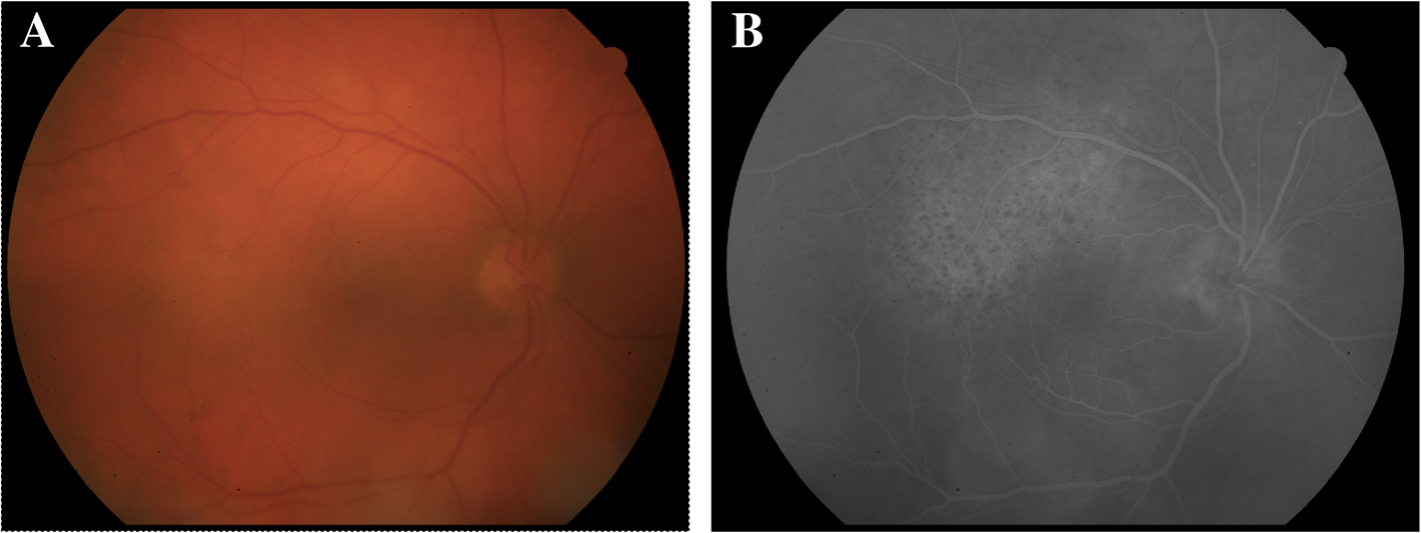
**Patient 2, pretreatment fundus imaging of the affected eye. (A)** Fundus photograph demonstrating broad areas of deep retinal whitening along the superotemporal and inferotemporal arcades. **(B)** Early phase fluorescein angiogram with leopard spotting in the superior macula.

Patient 2 was a 60-year-old Caucasian male with HIV infection (CD4+ T-cell count 600 cells/μL) who presented with decreased vision and a peripheral scotoma in the RE. Corrected acuity measured 20/63 in the RE and 20/16 in the LE. There were 1+ anterior chamber cells and vitreous cells in the RE. Fundus examination of the RE revealed an area of subtle retinal whitening along the superotemporal arcade, better appreciated as an area of 'leopard spotting’ with fluorescein angiography (Figure 2). The treponemal test was positive with a nonreactive RPR. He received a 14-day course of intravenous ceftriaxone (2 g/day); vision recovered to 20/25 in the RE with resolution of intraocular inflammation.

**Figure 3:**
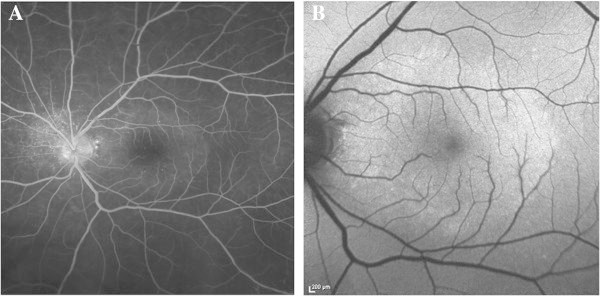
**Patient 3, pretreatment fundus imaging of the affected eye. (A)** Late phase angiogram demonstrating stippled, peripapillary hyperfluorescence, as well as a placoid area of hyperfluorescence in the macula. **(B)** Fundus autofluoresence image with increased autofluorescence in the macula.

Patient 3 was a 50-year-old Caucasian male with HIV infection (CD4+ T-cell count 830 cells/μL) who presented with decreased vision in the LE. Corrected acuity measured 20/20 in the RE and 20/60 in the LE. There were trace anterior chamber cells and 1+ vitreous inflammation in the LE. Fundus examination revealed small, white peripapillary retinal spots in both eyes and a placoid area of retinal whitening in the macula of the left eye that was particularly prominent in fundus autofluoresence (FAF) imaging (Figure 3). The treponemal test was positive with RPR titer of 1:4,096. He received 14 days of intravenous penicillin G (24 million units/day); visual acuity measured 20/25 in the left eye 3 months after treatment.

**Figure 4:**
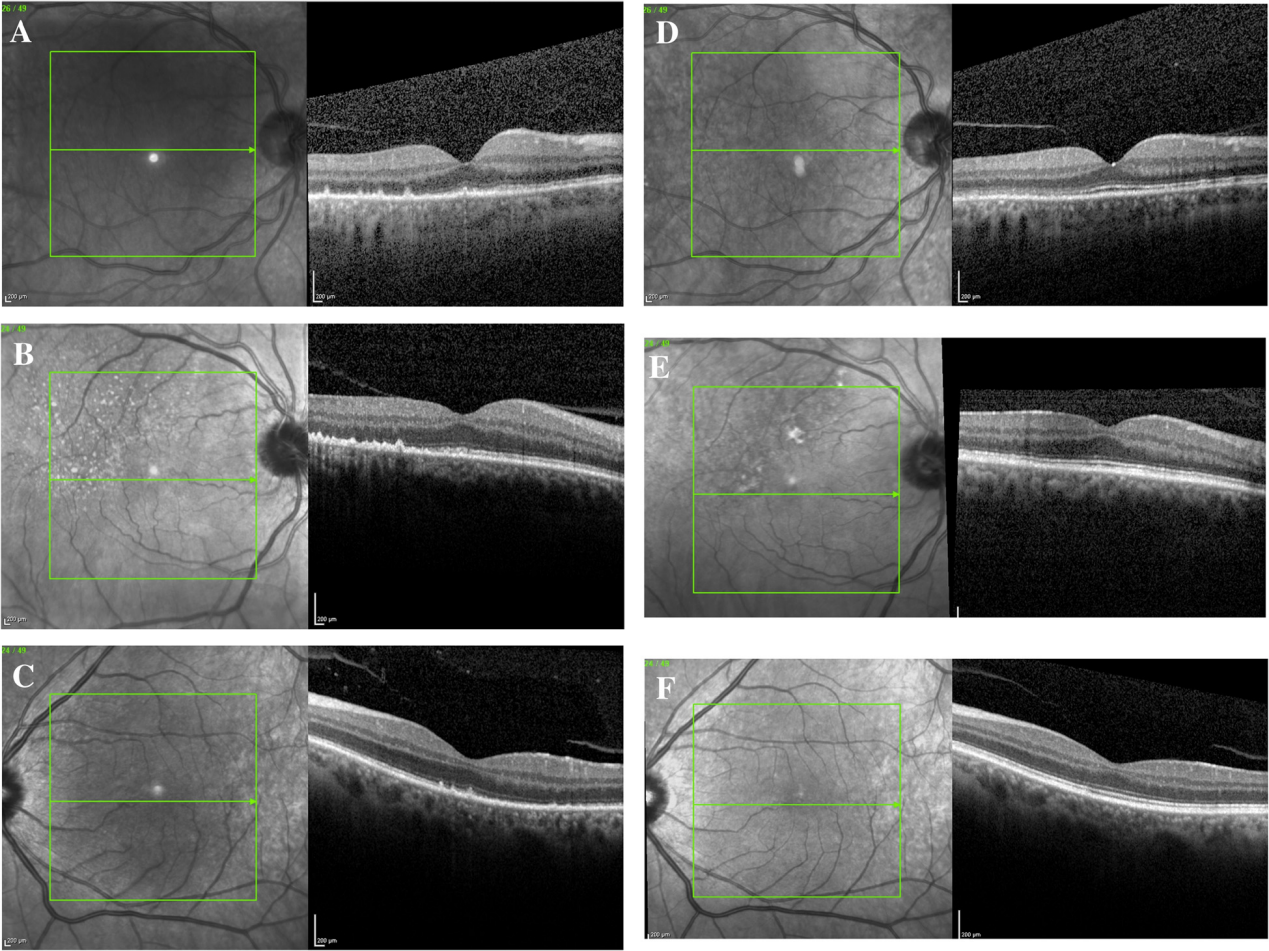
**SD-OCT images obtained from the affected eyes.** Spectral domain optical coherence tomography images **(A to C)** from the affected eyes of patients 1 to 3, respectively, prior to antibiotic treatment. The images demonstrate hyperreflective nodularity of the retinal pigment epithelium with overlying loss of the normal photoreceptor architecture. Spectral domain optical coherence tomography images **(D to F)** from the affected eyes of patients 1 to 3, respectively, 3 months after antibiotic treatment. The images demonstrate almost complete resolution of the RPE nodules with a more clearly defined photoreceptor inner segment-outer segment junction.

**Figure 5:**
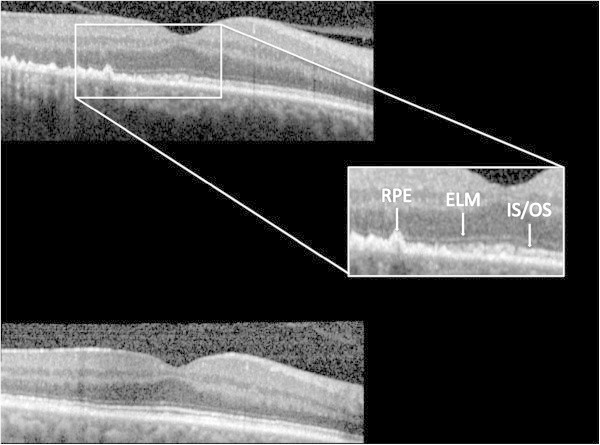
**Spectral domain optical coherence tomography images from the affected eye of patient 2.** The top image shows pre-treatment pathology, including (inset) retinal pigment epithelium (RPE) nodularity, disruption of the external limiting membrane (ELM), and loss of the photoreceptor inner segment/outer segment (IS/OS) band. The bottom images demonstrate resolution of these changes after antibiotic therapy.

SD-OCT images obtained from the eyes with ASPPC prior to antibiotic treatment showed several common findings (Figures 4, A-C and 5). There were scattered, focal nodules at the level of the RPE, corresponding to the yellow-white punctate lesions seen clinically. A loss of the photoreceptor inner segment-outer segment junction was seen overlying these RPE changes. Focal disruption of the external limiting membrane (ELM) was also noted in patients 1 and 2. Three months after treatment, the RPE nodules had largely resolved, the ELM was restored, and the inner segment-outer segment junction showed only mild disruption in each case (Figures 4, D-F and 5).

## Discussion

In all three patients, SD-OCT images demonstrated common features of (1) hyperreflective nodularity and thickening of the RPE in the area corresponding to the fundus lesion and (2) disruption and loss of the photoreceptor inner segment-outer segment junction overlying these RPE changes. No subretinal or interstitial fluid was seen. Despite visual improvement, subtle OCT changes persisted in the affected eyes, relative to the unaffected fellow eyes, 3 months after antibiotic therapy. It is unclear whether these mild abnormalities will continue to resolve with time.

Time domain OCT (TD-OCT), a lower-resolution predecessor to SD-OCT, first demonstrated hyperreflectivity and thickening of the subfoveal RPE-choriocapillaris complex in ASPPC [3,8,9]. A recent case series of SD-OCT findings in this disease reports nodular RPE thickening and photoreceptor disruption, as well as punctate hyperfluorescence in the choroid [10]. The authors also note transient subretinal fluid, which resolved before treatment, in about half of the eyes on initial presentation. We observed similar findings in the RPE and photoreceptor layers of our patients. The absence of subretinal fluid in our series may reflect lag time between initial onset of symptoms and presentation to our clinic. While patients 1 and 3 presented to our clinic about a week after symptom onset, patient 2 reported visual changes for about 2 months prior to his referral to our clinic. In the series by Pichi and colleagues, subretinal fluid was noted only in patients who had imaging done within the first 2 days of presentation; images from 1 week after presentation and later failed to demonstrate any subretinal fluid [10]. The images from our series also support the concept that subretinal fluid is a very early feature of ASPPC.

We also observed that, while two of the three patients in our series had HIV co-infection, there were no clear differences in clinical presentation, OCT findings, or response to treatment between these patients and the patient without HIV. Of note, both patients had been diagnosed with HIV more than 10 years ago and were on antiretroviral therapy with good immune recovery.

The pathophysiology of ASPPC is not completely understood. Gass et al. postulated that widespread dispersion of spirochetes may cause an inflammatory reaction at the level of the choroid-RPE-photoreceptor complex, resulting in the clinical appearance of the white placoid lesion and photoreceptor dysfunction [1]. Deposition of soluble immune complexes in the RPE has also been invoked in the pathophysiology [2]. Other imaging modalities may be helpful in understanding the pathogenesis of these fundus lesions. Indocyanine green angiography (ICGA) demonstrates hypofluorescence of the fundus lesions, suggestive of inflammation at the level of the inner choroid [3,8]. The choriocapillaris, then, may be the primary focus of the inflammatory process within the eye, with contiguous spread to RPE and then to the photoreceptors [8]. Some authors have postulated that ICGA hypofluoresence results from deposition of degraded material from the RPE and photoreceptor segments [11]. Similarly, FAF imaging in the eyes with ASPPC demonstrates increased autofluorescence within the lesions, consistent with accumulation of lipofuscin or photoreceptor outer segment remnants in the RPE [3,7]. The SD-OCT images in our series demonstrating RPE nodularity may be consistent with this theory. Further study is needed, both in the earliest phases of the disease, as well as in long-term follow-up.

In conclusion, SD-OCT imaging in patients with ASPPC demonstrate characteristic changes, including RPE deposits and disruption of the photoreceptor layers. These changes are consistent with an inflammatory process at the level of the choroid/RPE complex, with extension to the photoreceptors, and are largely reversible with appropriate antibiotic therapy. SD-OCT imaging of the chorioretinal lesions in the eyes with ASPPC may provide a better understanding of the pathophysiology of this particular type of syphilitic chorioretinitis.

## Abbreviations

ASPPC: acute syphilitic posterior placoid chorioretinitis; FAF: fundus autofluoresence; ICGA: indocyanine green angiography; IRB: Institutional Review Board; LE: left eye; RE: right eye; RPE: retinal pigment epithelium; RPR: rapid plasma reagin; SD-OCT: spectral domain optical coherence tomography; TD-OCT: time domain optical coherence tomography.

## Competing interests

The authors declare that they have no competing interests.

## Authors' contributions

BMB, JPD, TAO, NJB, and TGL designed and conducted the study. BMB collected and managed the data. BMB, JPD, NJB, and TGL analyzed and interpreted the data. BMB, JPD, TAO, NJB, JET, and TGL prepared, reviewed, and approved the manuscript.

## Authors' information

All authors are affiliated with the Division of Ocular Immunology at the Wilmer Eye Institute. JET is a professor of Ophthalmology and Epidemiology. JPD is an associate professor of Ophthalmology. BMB, NJB, and TGL are assistant professors of Ophthalmology. TAO is a Uveitis Fellow.
